# Digital Media Teaching and Effectiveness Evaluation Integrating Big Data and Artificial Intelligence

**DOI:** 10.1155/2022/1217846

**Published:** 2022-09-22

**Authors:** RuiYao Zhang

**Affiliations:** School of Literature and Journalism, Shandong University of Finance and Economics, JiNan 250014, China

## Abstract

With the development of digital media technology, its application in teaching and learning is becoming more widespread. Digital media technology helps present information in transmitting knowledge or skills, reduces cognitive load, and promotes understanding of knowledge. Evaluation of the effectiveness of digital media teaching has also become important. A scientific and reasonable evaluation of digital media teaching effectiveness can help teachers select digital media technology and grasp the amount, degree, and timing of digital media use to change teaching effectiveness. This paper proposed using a combination of big data and artificial intelligence methods to evaluate the effectiveness of digital media teaching methods using the RBF neural network model. The digital media teaching effectiveness evaluation was used as the input variable of RBF, the degree of digital media effectiveness was the output variable and the neural network was trained through the collected sample data. The research results showed that the RBF neural network model proposed in this paper has a strong generalization and extension ability in evaluating digital media teaching effectiveness, providing a new way to evaluate digital media teaching effectiveness.

## 1. Introduction

The development of digital media technology opens up a wide range of opportunities for education, resources, and development circumstances. It also revolutionizes contemporary educational technology and further influences changes in educational theory, concepts, techniques, and approaches. Digital media teaching not only plays the role of teaching aid as a kind of media but has also been integrated into modern educational technology and participates in the whole teaching process [1]. Digital media teaching is no longer limited to the audio-visual category but presents new characteristics such as multimedia, hypersexualization, networking, and interactivity. The introduction of digital media aids in classroom teaching and can stimulate students' interest in learning to a greater extent, promoting students' understanding and mastery of knowledge. It improves students' ability to break through difficult points, broadens their thinking, changes their learning attitudes, and improves the efficiency of classroom teaching and the quality of imparted knowledge so that students' learning performance has improved. Digital media teaching advances teaching objectives by providing a way of knowledge transmission that is easy for students to understand and accept and motivates students to learn through vivid multimedia expressions, effectively improving teaching effectiveness. The development of digital media technology has provided teachers with a broader range of choices and applications of teaching media. Many problems have arisen in the face of a wide variety of digital media with different functions, which are more and more frequently used in teaching. For example, teachers are obsessed with the new and ignore the role of traditional teaching media. Teachers blindly choose digital media and pursue the diversification of external application forms, resulting in the phenomenon of using digital media too much and too widely. Some educators ignore student-teacher contact in favor of concentrating solely on media use. The overemphasis threatens teachers' dominance and students' primacy in digital media technologies in the classroom.

Modern educational theory believes that students are the main body of teaching activities, and any teaching means, teaching tools, and teaching media should be used to highlight the primary position of students, and digital media teaching is no exception. Digital media should be a tool for students to receive, discover, explore new information, and finally, master knowledge to the best of their ability, and a helper for students to learn, not just for teachers to explain and demonstrate. It is also an essential basis for the digital media teaching methods teachers choose and use. It can be seen that how to help correctly assess the effectiveness of digital media teaching and achieve reasonable use of digital media teaching is an urgent problem we need to solve.

## 2. Literature Review

### 2.1. Digital Media Teaching and Its Effectiveness Evaluation

#### 2.1.1. Digital Media Teaching and Learning

“Media” refers to the material entity that transmits information symbols [2]. A carrier that carries information, an entity that stores and transmits information, and a tool placed into the communication process are all examples of media. Communication media include language, books, newspapers, radio, film, television, computers, and the Internet [3].

Communication media that have been digitally encoded are known as “digital media”. Computers allow for the creation, browsing, distribution, modification, and storage of digital material, which includes e-books, digital photos, movies, Internet pages, data, and databases [4]. The digitization of information in many media has helped the growth of digital media and made it possible for information in various media to be converted to digital form. The blending of various media is now possible. The technological bar between different media is being lowered due to the ongoing specialization, pasteurization, and civilianization of digital media, making it simpler to aggregate the interchange of many types of information [5].

When media convey information for instructional purposes, they are called instructional media. Instructional media is a general term for numerous instructional materials and refers to the carriers or intermediaries that convey educational information in educational and teaching activities. [6].

Instructional media are the means or tools for presenting information in the process of transmitting knowledge or skills. The term “digital media teaching” refers to the process of teaching that takes into account the characteristics of the learning objectives and learning materials through the design of the lesson, the thoughtful selection and application of digital media technology; the organic integration of traditional teaching methods; and joint participation in the entire teaching process with a variety of media information roles for the students, as well as the development of an appropriate teaching process structure, to ensure that students learn in the most effective way possible [7].

The emergence of digital media technology makes teaching both face-to-face and distant classroom teaching, bringing a revolutionary breakthrough to classroom teaching.

Teaching with digital media has gradually replaced the integrated use of multiple teaching media. Digital media teaching requires teachers to have professional media use techniques, courseware system level, and students to give full play to learning initiatives.

#### 2.1.2. Evaluation of the Effectiveness of Digital Media Teaching

The study of digital media teaching effectiveness focuses on applying digital media technology to improve teaching effectiveness. Teaching effectiveness refers to the teacher's ability to enhance teaching effectiveness through classroom activities, so students gain, improve, and progress academically. This is manifested in the cognitive aspect of students, which enhances their understanding of knowledge; the emotional aspect, which stimulates interest in learning; and the outcome aspect, which enhances academic performance. How to use digital media devices and technologies, apply digital media technologies to teaching, and organize and integrate multiple digital media technologies affects teaching effectiveness [8]. Research shows that digital media teaching should be combined with the corresponding principles to change the teaching effect by grasping the amount, degree, and timing of digital media use. The use of digital media technology can increase student engagement in learning, solve problems with instruction, and visualize abstract information and its corporatization to enhance the effectiveness of classroom instruction [9].

Evaluation of digital media teaching effectiveness can identify the influencing factors of digital media teaching. The indicators of teaching evaluation can classify the level of the influencing factors by level and formulate effective measures to influence and optimize digital media teaching from both micro and macro aspects. The evaluation principles of digital media teaching effectiveness follow measurability, systematization, scientificity, adaptability, and guidance. Its evaluation index model should contain evaluation criteria for students, teachers, teaching courseware, and teaching platforms, covering the evaluation of the teaching plan scheme, teaching instruction process, and teaching instruction effect.

### 2.2. Big Data and Artificial Intelligence and Their Applications in Effectiveness Evaluation

#### 2.2.1. Big Data and Artificial Intelligence

The discipline of artificial intelligence has a history of more than 50 years and is internationally recognized as one of the cores of contemporary high technology [10]. Artificial intelligence computers replicate intelligent human behavior to complete computer tasks like picture identification, language recognition, and other specific intelligent activities [11]. Because artificial intelligence is a broad field, research into its applications has penetrated many different fields and disciplines with impressive results. The development of artificial intelligence is aided by mixing and utilizing the research and advancements made in other fields, which introduces fresh viewpoints and concepts to the discipline. Born in the 1950s, bionics is a niche field that combines biological science and engineering technology to enhance current machinery and processes or even develop entirely new ones by learning from, copying, and reproducing the structure, function, and operating principles of biological systems [12]. Inspired by the mechanisms of biological evolution, researchers have proposed many new approaches for solving artificial intelligence problems. At the same time, with the gradual understanding of the way human and animal brains cognize and work in neurology, biology, and cognitive science, as well as advances in computer technology, methods from mathematics, physics, and information processing have been combined to study the composition and working principles of the human brain's neural networks and to build simplified abstract models, resulting in a new research field called artificial neural networks [13].

An artificial neural network is a large-scale, nonlinear adaptive system that mathematically represents the human brain and its activities in theory. It comprises several processing units that are intelligently coupled to each other [14]. Neural networks are a method of data classification based on a distance metric [15]. Neural networks were created inspired by knowledge of the physiology of tissues [16]. They are composed of interconnected, identical units (neurons). The interconnections can transmit either enhancement or inhibition signals between different neurons. Enhancement or inhibition is achieved by adjusting the weight coefficients of the interconnections. Neural networks can perform classification and regression under supervised and unsupervised learning conditions, which is achieved by appropriately adjusting the weight coefficients [17]. One converge the output of a neural network to the correct target value using a weight coefficient adjustment mechanism.

The artificial neural network model has many advantages, such as less human intervention, high model accuracy, strong self-learning capability, and associative storage function, etc., [18]. It consists of many processing units extensively interconnected, which allows some simulation of biological systems. However, it also has excellent disadvantages, such as high requirements for data samples, inability to process and describe fuzzy information; and black box nature, i.e., its output process and results do not have interpretability [19]. Therefore, artificial neural network technology is often applied to big data technology [20].

In evaluating the effectiveness of digital media teaching, its evaluation indexes are random and dynamic, and there is no one-to-one functional relationship between the elements of evaluation indexes and the results, so neural networks have more significant advantages for effectiveness evaluation.

#### 2.2.2. RBF Neural Network and Its Learning Algorithm

The RBF neural network is a feed-forward neural network with excellent performance. Compared with other structural neural networks, it can approximate any nonlinear function with arbitrary accuracy and has the ability of global approximation. The structural parameters can be separated for learning, the convergence speed is fast, and it has high generalization ability. Therefore, this paper chooses the RBF neural network to model the effectiveness of digital media teaching.

**Figure 1:**
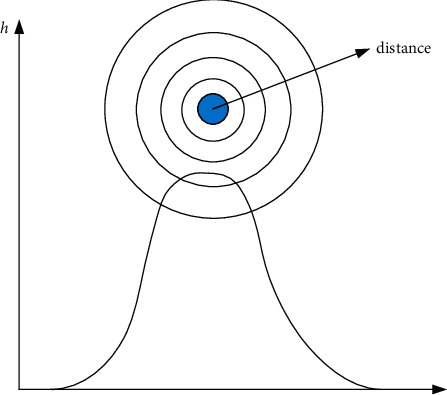
The RBF function diagram.

*(1) RBF Neural Network Structure.* The genuine multivariate difference problem, a high-dimensional space curve-fitting problem, is where the radial basis function was initially used [21]. The Euclidean distance between any point *x* and a center *c* in the space is typically used to define the radial basis function as a monotone function. Since, it only has a local impact, as demonstrated by formula (1), when *c* is far from *c*, the value of the function is small [22]. The effect of this function is shown in Figure 1.

(1) $$\begin{array}{c} h\left(x\right)=\exp\;\left(-\frac{{\left(x-c\right)}^{2}}{r^{2}}\right)\text{.} \end{array}$$

**Figure 2:**
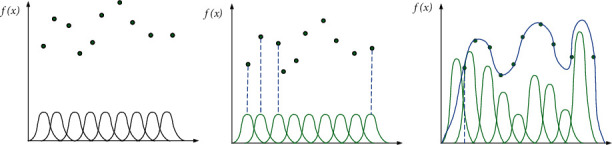
The RBF interpolation function construction diagram.

The primary purpose of the radial basis function is to address the issue of multivariable interpolation [21]. Taking two-dimensional sample points as an example, the construction method of the RBF interpolation function is shown in Figure 2. The first subgraph represents the initial distribution of sample points, the second subgraph represents placing a basis function on each sample, and the third subgraph represents the constructed RBF interpolation function curve. [23].

RBF interpolation is constructing a function *F*(*x*)=∑_*i*=1_^*N*^*w*_*i*_*φ*_*i*_(‖*x* − *x*_*i*_‖) whose curve passes through all known sample points. Without loss of generality, we only consider the case where the dimension of the output space is *m* = 1.

Given N sample points (*x*_1_, *y*_1_), (*x*_2_, *y*_2_), ⋯(*x*_*N*_, *y*_*N*_), substitute *y*_*i*_=*F*(*x*_*i*_), *i*=1,2, ⋯, *N* into the equation above, and let *φ*_*ji*_=*φ*(‖*x*_*j*_ − *x*_*i*_‖) get

(2) $$\begin{array}{c} \left[\begin{array}{cccc} \varphi_{11} & \varphi_{12} & ... & \varphi_{1N}\\ \varphi_{21} & \varphi_{22} & ... & \varphi_{2N}\\ ... & ... & ... & ...\\ \varphi_{N1} & \varphi_{N2} & ... & \varphi_{NN} \end{array}\right]\left[\begin{array}{c} w_{1}\\ w_{2}\\ ...\\ w_{N} \end{array}\right]=\left[\begin{array}{c} y_{1}\\ y_{2}\\ ...\\ y_{N} \end{array}\right]\text{.} \end{array}$$

Write it in matrix form Φ*W*=*Y*, and it gets *W*=Φ^−1^*T*.

After using RBF to build artificial neural networks, Broomhead and Lowee created the RBF neural network. Pattern categorization and function approximation problems are typically solved with RBF neural networks. Cover's theorem, which states that it is possible to solve classification problems for pattern problems in the high data space that are difficult to address in the low dimensional space, mathematically ensures the rationality of the RBF neural network topology. This explains why RBF neural networks typically have high dimensionalities. The network's performance is directly correlated with the dimensionality of the hidden layer space; that is, the larger the dimensionality, the higher the approximation accuracy. The issue is, however, that higher dimensionality also means increased nonlinearity complexity.

**Figure 3:**
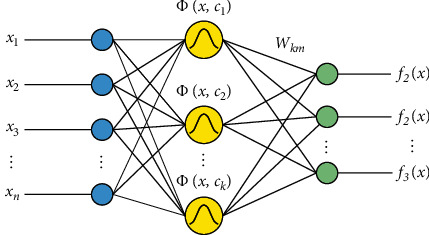
The structure of the RBF neural network.

The input, hidden, and output layers are the three levels that make up the most fundamental RBF neural network. Each layer serves an entirely different purpose. The hidden layer performs a nonlinear transition from the input space to the hidden space, typically with a high dimensionality; the output layer is linear and reacts to the activation signal acting on the output layer. The input layer connects the network to the outside world [24].  Figure 3 depicts the structure of a three-layer RBF neural network with *n* input nodes, *k* hidden layer nodes, and *m* output nodes.

Where *x*=[*x*_1_, *x*_2_, ⋯,*x*_*n*_]^*T*^ is the input loss, Φ=[*ϕ*(*x*, *c*_1_), *ϕ*(*x*, *c*_2_), ⋯, *ϕ*(*x*, *c*_*k*_)] is the network hidden layer output matrix, *c*_*i*_ is the *i-th* hidden layer node position, *i*=1,2, ⋯, *k*; *W*_*km*_=[*w*_1_, *w*_2_, ⋯,*w*_*k*_]^*T*^ is the network output weight matrix, *w*_*i*_=[*w*_*i*1_, *w*_*i*2_, ⋯,*w*_*im*_]^*T*^, *F*(*x*)=[*f*_1_(*x*), *f*_2_(*x*), ⋯,*f*_*m*_(*x*)]^*T*^is the network output vector.

The activation functions of the hidden layer are radial basis functions, which usually satisfy Micchelli's theorem [25]. Micchelli's theorem states that as long as each sample point is a mutually distinct point in the space, the resulting interpolation matrix Φ is nonsingular [26]. Three radial basis functions occupy an important position in the RBF neural network.

(1) Multiquadratic function. Whose function expression is shown in the following formula:
  (3) $$\begin{array}{c} \phi \left(x,c_{i}\right)={\left(x^{2}+c_{i}^{2}\right)}^{1/2}i=1,2,\cdots ,k\text{.} \end{array}$$
(2) Inverse multiquadratic function. The function expression is shown in the following formula:
  (4) $$\begin{array}{c} \phi \left(x,c_{i}\right)=\frac{1}{{\left(x^{2}+c_{i}^{2}\right)}^{1/2}}\text{.} \end{array}$$
(3) Gauss function. Its functional expression is shown in the following formula:

(5) $$\begin{array}{c} \phi \left(x,c_{1}\right)=\exp\;\left(-\frac{{\left\|x-c_{i}\right\|}^{2}}{\sigma^{2}}\right),i=1,2,\cdots ,k\text{,} \end{array}$$

where *σ* is the hidden layer center width.

*(2) The RBF Neural Network Learning Algorithm.* The hidden unit's activation function in an RBF neural network typically has a local acceptance domain. In other words, the hidden layer nodes will only respond meaningfully when the input falls into a narrowly defined region in the input space.

The radial basis function is utilized as the “base” of the hidden layer unit to create the hidden layer space in an RBF neural network. This mapping relationship is determined together with the radial basis function centroid. The network's output is the linear weighted sum of the hidden nodes since the mapping between the hidden space and the output space is linear. As can be seen, the network's overall input to output mapping is nonlinear, although the hidden layer to output mapping is linear.

The development of the hidden layer, which entails determining the hidden layer's number of nodes and its center and width, is the crucial and challenging step in creating an RBF neural network. The complexity and generalizability of the network are influenced by the number of nodes in the hidden layer. Imagine there are not enough nodes. In that instance, the network model will be constrained, limiting the network's capacity for generalization. On the other hand, if too many nodes exist, the network's capacity for generalization will also be diminished. The most important decision is where to put the hidden layer's center. When the center placement is incorrect, the RBF neural network cannot realize the transition from the nonlinear input space to the linear output space, accurately reflecting the accurate division of the input sample space. The RBF neural network's capacity for classification is also significantly influenced by the width of the hidden layer's center. When the width is too broad, the line separating classes blurs, and classification accuracy suffers; when the width is too small, the kernel function's coverage area is limited, and the network's generalization capacity suffers.

The more common RBF neural network learning algorithms are randomly selected fixed center, self-organized center selection, and supervised center selection according to the different methods of determining the center of the basis function [27].

*(i) Randomly Selected Fixed Centers*. The simplest method assumes that the activation function defining the hidden layer nodes is a fixed radial basis function. The location of the centers can be randomly selected from the learning dataset, which is suitable if the learning samples are distributed in a way typical of the current problem. In particular, a radial basis centered at ci is defined as follows:

(6) $$\begin{array}{c} \phi \left(x,c_{i}\right)=\exp\;\left(-\frac{k}{d_{\max}^{2}}{\left\|x-c_{i}\right\|}^{2}\right),i=1,2,\cdots ,k\text{,} \end{array}$$

where *k* is the number of hidden layer nodes, and *d*_max_ is the maximum distance from the selected center. It can be seen by Formula (6) that the widths of the hidden layer nodes are all fixed, as shown in Formula (7). This formula ensures that no radial base is too sharp or too flat. Too sharp or too flat should be avoided as much as possible.

(7) $$\begin{array}{c} \sigma =\frac{d_{\max}}{\sqrt{2k}}\text{.} \end{array}$$

In this approach described above, the only learning parameter that needs to be determined is the linear weight *W* of the output layer, and a straightforward approach is a pseudo-inverse method, as shown in the following formula:

(8) $$\begin{array}{c} W=G^{+}Y\text{,} \end{array}$$

where *Y* is the desired output vector in the learning set. The matrix is the pseudo-inverse of the matrix *G*^*+*^, and *G* is defined as shown in the following formula:

(9) $$\begin{array}{c} G=\left\{g_{gi}\right\}\text{.} \end{array}$$

Among them,

(10) $$\begin{array}{c} g_{qt}=\phi \left(x_{q},c_{i}\right)=\exp\;\left(-\frac{k}{d_{\max^{2}}}{\left\|x_{q}-c_{i}\right\|}^{2}\right),q=1,2,\cdots ,N,i=1,2,\cdots ,k\text{.} \end{array}$$

In the above equation, *x*_*q*_ is the *q-th* input vector in the learning sample, and *c*_*i*_ is the *i-th* center.

*(ii) Self-organizational Selection of Centers*. The main drawback of the fixed-center approach is that a large learning set is required to achieve satisfactory performance. One way to overcome this drawback is to use a hybrid learning process consisting of two distinct phases: a self-organized learning phase and a supervised learning phase. The purpose of the first stage is mainly to estimate a suitable position for the hidden layer nodes, while the second stage is to calculate the output weights of the network based on the node positions estimated in the first stage, completing the design of the RBF neural network. The representative algorithms of this method include the K-means clustering algorithm, the GCLS (Generalized Competitive Learning Scheme) learning algorithm, and so on. Taking the GCLS learning algorithm as an example, its learning algorithm process is as follows:

Initialization determines the number of initially hidden layer nodes *k*, the initial hidden layer node parameters *c*_*i*_ and *σ*_*i*_*, j* *=* 1, 2,* ..., k*.

Randomly select the input vector *x*_random_ from the learning sample set, calculate the Euclidean distance of this sample to each center according to formula (11), and find the winning node according to the nearest distance criterion, i.e., the nearest node *c*_*nearest*_, corresponding to the distance *d*_min_.

(11) $$\begin{array}{c} d_{i}=\left\|x_{random}-c_{i}\right\|,i=1,2,\cdots ,k\text{.} \end{array}$$

If *d*_min_*≥ δ* (*δ* is a positive constant), the following formula is used to add hidden layer nodes to the network.

(12) $$\begin{array}{c} k=k+1;c_{k}=x_{random}\text{.} \end{array}$$

Instead, the following formula is used to update the hidden layer parameters.

(13) $$\begin{array}{c} c_{i}\left(t\right)=\left\{\begin{array}{cc} c_{i}\left(t-1\right)+\eta \left(x_{random},c_{i}\left(t-1\right)\right)\text{,} & Ifc_{i}win\text{,}\\ c_{i}\left(t-1\right)\text{,} & Other\text{,} \end{array}\right. \end{array}$$

where *η* is the step size and *t* is the number of learning times.

The above steps are repeated until the error reaches the standard or the maximum number of learning opportunities is required. After that, the gradient descent method can be used to learn the correction of the weights.

A limitation of this method is that it can only reach the optimal local solution that depends on the initial values of the selected centers because the initial values of some centers may be located in the region of sparse data points in the input space. The final result may be an unnecessarily large network structure and a waste of computational resources.

*(iii) Supervised Selection of Centers.* In this method, all free parameters of the network undergo a supervised learning process. The method uses an error-correction learning process, which can be easily used with gradient descent. There are several points to note when using this method: the cost function is convex for the weights but nonconvex for the center location and width, so the center location and width may fall into local minima in the parameter space; different learning steps should be chosen when updating the network parameters; and unlike the BP algorithm, there is no error back propagation in the gradient descent method of RBF neural networks.

These are the most commonly used RBF neural network learning algorithms. A good algorithm can improve network performance while saving network resources. Based on the above several ideas, many scholars have studied RBF neural network learning algorithms while improving them for their different shortcomings, so studying RBF neural network learning algorithms has reached a new level. Researchers have combined center assignment and weight updating, for example, using unsupervised clustering algorithms to determine centers and widths, and supervised learning algorithms to determine weights.

**Figure 4:**
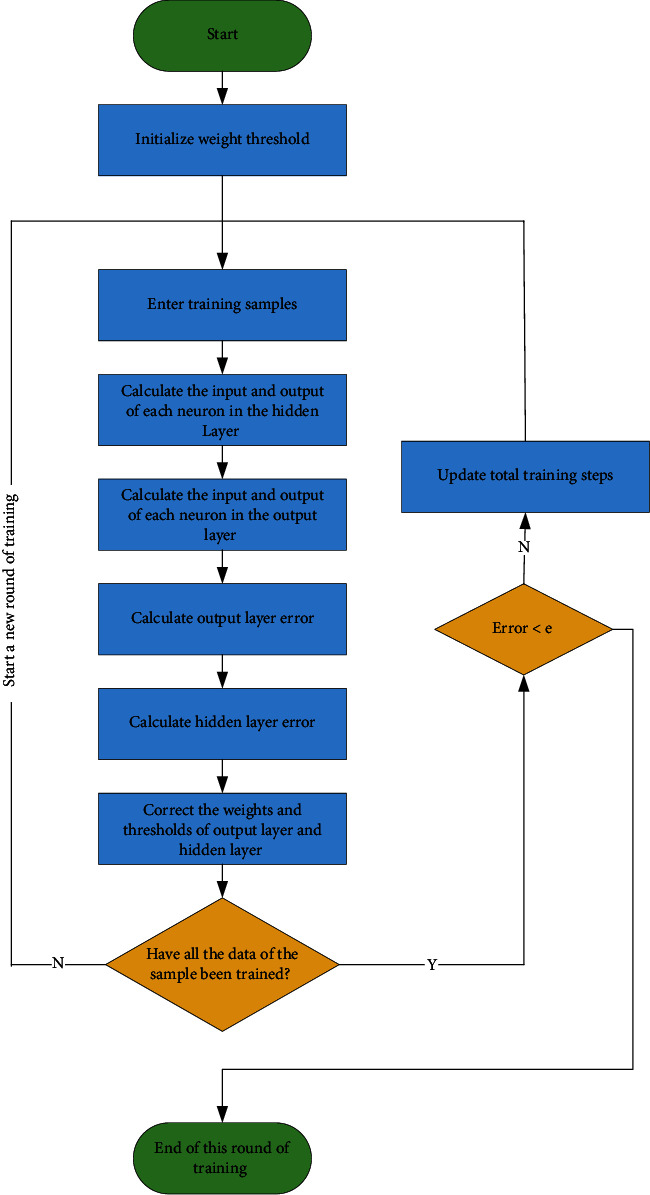
The RBF neural network training process.

The RBF neural network has the unique best approximation property, no local minima problem, vital input and output mapping functions, good classification ability, and fast convergence of the learning process. However, when the number of training samples increases, the number of hidden layer neurons of the RBF network is high, which increases the complexity, and the structure is too large. RBF neural network training first initializes the weight threshold and then conducts training on the input training samples. The input and error of each layer are calculated, respectively, until all data of the samples are trained, the error is less than *e*, and the training ends. The specific process is shown in Figure 4.

## 3. Method

### 3.1. Constructing the Evaluation Index System

#### 3.1.1. Basic Principles for Constructing the Evaluation Index System of Digital Media Teaching Effectiveness

1. *The principle of Completeness*. The principle of completeness means that the developed index system can reflect, reproduce, and cover all aspects that affect the effectiveness of digital media in a comprehensive, systematic, and essential way. Only when the index system has the overall completeness can it reproduce and reflect the effect of digital media technology comprehensively and without omission and provide comprehensive teaching information to evaluate digital media effectiveness. However, the evaluation indexes should simultaneously catch the key factors and main contradictions.
2. *Independence Principle*. The independence of indicators refers to the fact that indicators of the same level shall not be duplicated, crossed, or interpolated; logically speaking, they shall not have the same relationship, cross-relationship, or cause-and-effect relationship but must be juxtaposed. Therefore, it is essential to ensure the independence of indicators.
3. *Harmony Principle*. Harmony of the indicators means that the indicators must be connected, coordinated, harmonious, and compatible and not contradict each other. Only if the index system has the principle of harmony can it accurately measure the various dimensions of digital media effectiveness and avoid contradictions between dimensions.
4. *The principle of Measurability*. The measurability of indicators refers to the nature of the content of the indicator system that can be expressed in a functional language and measured by specific means to obtain information and conclusions to make the abstract goal concrete. Evaluation indicators' measurability ensures that each can be measured accurately and compared with other indicators. If the principle of measurability is missing, it is not easy to ensure the precision and accuracy of evaluation indicators.
5. *The Principle of Dynamism*. Actual digital media teaching is a dynamic process in which the elements change from time to time, which may lead to many unknown effects, requiring us to establish an index system that is not always used. It needs to be constantly adjusted and amended according to the dynamics of the digital media teaching process as the research progresses to ensure the evaluation's correctness, scientificity, and objectivity.

#### 3.1.2. Constructing the Evaluation Index System of Digital Media Teaching Effectiveness

This paper collects digital media teaching effectiveness evaluation elements based on literature and a questionnaire survey. Through further generalization of the evaluation elements of digital media teaching effectiveness, it got the evaluation index system of digital media teaching effectiveness. The first-level indicators include five dimensions: teaching requirements, teaching process, media quality, teacher skills, and student learning; and each first-level indicator continues to be subdivided into five specific indicators, making up a total of 25 indicators.

#### 3.1.3. Creating the Digital Media Teaching Evaluation Grades Using the Delphi Method

In order to accurately measure the digital media teaching effectiveness grade, this paper uses the Delphi method to measure the digital media teaching effectiveness of the prototype objects. Experts evaluate the effectiveness of digital media instruction back-to-back.

### 3.2. Constructing the RBF Network

#### 3.2.1. Determining the Topology of the Network

The selection of the neural network structure model is critical, and the proper selection of the model can significantly reduce the number of network training and ensure high network learning accuracy. The RBF network is suitable for solving the classification evaluation problem. Since, this paper is to solve the digital media teaching effectiveness evaluation problem, the RBF neural network was selected as the evaluation model. The network structure, mainly, contained the connection method, network levels, and the number of nodes in each level, i.e., the number of neurons in the explicit input layer, implicit layer, and output layer. The problem of evaluating the effectiveness of digital media teaching can be seen as a nonlinear mapping of input values (digital media teaching methods) to output values (digital media teaching effectiveness levels).

According to the evaluation index, the input layer of RBF had 25 nodes. There were five evaluation categories. Each level corresponds to one category of samples, so the radial basis function nodes of the hidden layer of RBF were five, each corresponding to one category. The output layer was one node that gave the evaluation results. Next, the network parameters were initialized. There were two categories of network parameters for RBF, the first category was the center and variance of the hidden layer radial basis function nodes, and the second category was the output layer weights. Before using L-M iteration, both types of parameters need to set initial values. This paper used the K-mean clustering algorithm to determine the centers and variances of the hidden layer nodes. The basic idea of the K-mean clustering algorithm is to minimize the total intraclass distance (the sum of the sample-to-center distances in each class).

(14) $$\begin{array}{c} E=\overset{k}{\underset{j=1}{\sum }}\underset{x_{i}\in w_{j}}{\sum }{\left\|x_{i}-m_{j}\right\|}^{2}\text{.} \end{array}$$

The basic steps of the k-means clustering algorithm are as follows:

First, initializing the number of clusters *k*, the number of iterations *N*, the minimum deviation *δ* of successive iterations, the current generation *t* *=* *0*, and randomly selecting *k* centers (usually the first *k*) *m*_*tj*_.

Second, making *t* *=* *t* *+* 1, assign *x*_*i*_ to the class represented by its nearest center *m*_*j*_^*t*^; Third, calculating the new center *m*_*j*_^*t*+1^ and the performance index *E*(*t* *+* 1) after the assignment;

(15) $$\begin{array}{c} m_{j}^{t+1}=\frac{1}{N_{j}}\underset{x_{i}\in m_{j}^{l}}{\sum }x_{i}\text{.} \end{array}$$

Fourth, if *t* *>* *N or ||E*(*k* *+* 1) − *E*(*k*)||*<δ* stop, otherwise turn 2, continue.

The output layer weights are determined as random numbers uniformly distributed between intervals (−1, 1). Regarding the parameters of the L-M algorithm, *μ*_1_ = 0.1, *μ*_2_ = 1.0, the number of iteration steps is taken as 5000, and the error accuracy is taken as 10^−2^.

#### 3.2.2. Collecting Sample Data

The good or bad final result of artificial neural network training, reflecting the network's performance, is closely related to the selected training samples. A good training sample should focus on the number and quality of samples.

In this paper, the input data of digital media teaching rating indexes were obtained using a questionnaire. The questionnaire survey method had vital openness in time and space, broad dissemination, interactivity, and ease of data collection and management while covering personalized communication and convenient data statistics and analysis functions.

**Figure 5:**
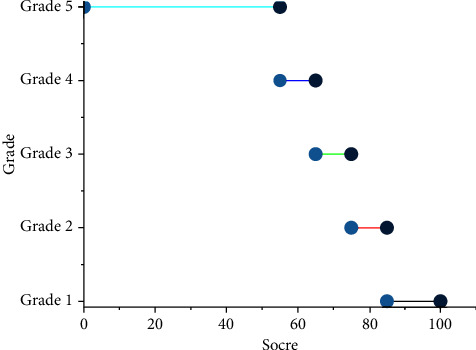
Score rating.

For the desired output index, this paper used the Delphi method for comprehensive scoring to evaluate the effectiveness of the digital media teaching method of the course. The Delphi method is essentially a feedback anonymous correspondence method. The general process is: after obtaining the opinions of experts on the problem to be predicted, it is organized, summarized, counted, and then anonymously fed back to each expert, again soliciting opinions, then focused, and fed back again until a consensus is obtained. According to the digital media teaching method, experts used the Delphi method to score the effectiveness of the course's digital media teaching and divided the scores into five levels. The scores of each level are shown in Figure 5. In this paper, 860 courses in a university were selected for the study, and the effectiveness evaluation indicators of each course in terms of teaching requirements, teaching process, media quality, teacher skills, and student learning were collected and asked to be evaluated by experts using the Delphi method, of which the first 75% were used for the training of the RBF network and the last 25% for testing the network generalization ability.

#### 3.2.3. Processing of Sample Data

Before inputting the data sample to the RBF network, the data should be normalized. The obtained data and the range of input values of the sample indicators vary in size. There will be significant differences in the values obtained for different indicators, so the data obtained for each indicator must be normalized first to prevent the significant value information from swamping the small value information. Normalization is to normalize the range of input values of indicators to between [0, 1] by operation, and the most commonly used are the exponential function method, the maximum-minimum method, etc. This study used the maximum-minimum method to normalize the data samples. This method is a linear transformation of the data, which can better retain the originality of the data and will not distort the data information. The input and output data were normalized to values between [0, 1], and the transformation equation was entered as follows:

(16) $$\begin{array}{c} \chi_{i}^{*}=\frac{\chi_{i}-\chi_{\min}}{\chi_{\max}-\chi_{\min}}\text{,} \end{array}$$

where *x*_*i*_ is the input data, *x*_min_ is the minimum value in the input data, and *x*_max_ is the maximum value in the input data.

## 4. Results

**Figure 6:**
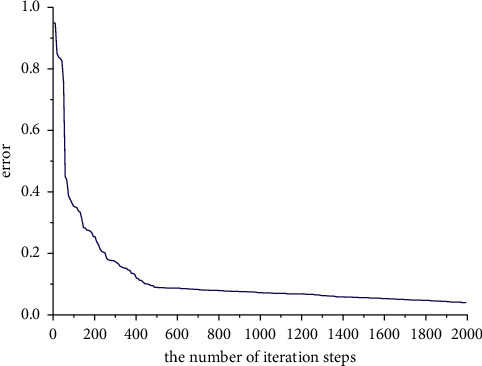
The RBF training process.

The sample data from the training set is submitted to the RBF network for training to approximate the complex mapping relationship between evaluation metrics and various evaluation results. The network converged after several iterations. The dynamic curve of the approximation error decreased with the number of iteration steps, as shown in Figure 6. The RBF neural network training results shows that as the training iterations proceed, the early convergence is faster, and the error decreases rapidly. The latter's training error decreases less until the number of training sessions reaches about 1800, and the training error tends to be smooth.

**Figure 7:**
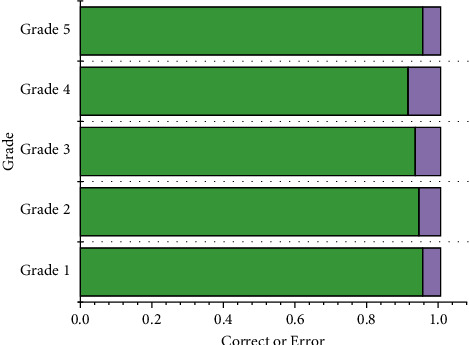
The rate of correct and error in the test set.

The original intention and destination of designing neural network training is applied. Whether or not the trained network can be applied depends on whether the network has light generalization and promotion ability. Generalization capability is the key criterion for measuring the merit of neural network evaluation methods. A network with good performance in the training phase is a failed and unusable model if it has no generalization ability. In this paper, the maximum test error was 0.0513 among all test samples, of which 198 samples were judged correctly. Therefore, the evaluation of the RBF network is correct up to 92%. The rate of correct error in the test set was shown in Figure 7.

**Figure 8:**
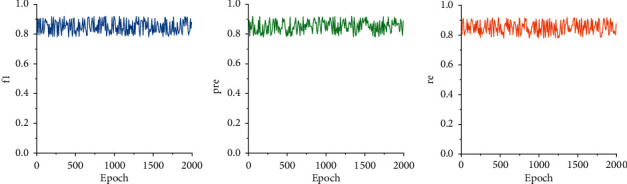
The F1, Pre, and Re of the RBF.

The training results of the RBF model's F1 score, precision (Pre), and recall (Re) are shown in Figure 8.

The validation results show that the improved RBF neural network and the evaluation model of teaching effectiveness had strong generalization ability. Thus, this study provided a new way to solve the problem of evaluating the effectiveness of digital media teaching.

## 5. Discussion

There are many factors affecting the effectiveness of digital media teaching, and this paper, by establishing an evaluation index system, only contains specific influencing factors, which cannot fully reflect all the situations of digital media teaching. The data collection on the effectiveness of digital media classroom teaching is also not extensive, and the results can only reflect the level of digital media teaching effectiveness of the sample to a certain extent. The scope of sample collection and sample size should be expanded subsequently to make the results more universal.

With the use of new technologies and new teaching concepts, digital media teaching will also produce profound changes. Whether these changes in digital media technology can effectively promote teaching and learning requires determining the influencing factors and doing a lot of investigation and practical research. Using digital media technology in teaching is also a process of constantly discovering the advantages of digital media technology, discovering the shortcomings of digital media technology, correcting the shortcomings, and optimizing it.

Although the RBF neural network model can improve the efficiency of digital media teaching effectiveness evaluation and the objectivity and fairness of evaluation results, the universality of the RBF neural network model is poor. Therefore, the next problem is how to improve the generality of the evaluation model and make it universally applicable to various types of media teaching effectiveness evaluation, as well as how to extend the model to other than traditional media teaching effectiveness evaluation.

## 6. Conclusions

The evaluation model of digital media teaching effectiveness using the RBF neural network is operable and provides another useful and convenient tool for the scientific evaluation of teaching quality. This study collected the evaluation elements of digital media teaching, constructed the evaluation index system of digital media teaching, and collected the effect of digital media teaching using by the Delphi method. The research used an RBF neural network model to train and validate the evaluation indexes and evaluation effects, including sample selection, processing, training of the created RBF neural network model, and the whole process of using the trained neural network to measure and compare the measurement results with the expert evaluation results. Furthermore, the established RBF neural network based on the LM algorithm training speed advantage was prominent. It could meet the error accuracy requirements, and the error was small. The data measurement using the trained network model also achieves results very close to those of the expert evaluation. This paper introduces big data and artificial intelligence technology to evaluate digital teaching media teaching effectiveness. It attempts to evaluate and predict the effectiveness of digital media teaching using a quantitative approach. The prediction based on the RBF neural network model not only ensures the objectivity and accuracy of the evaluation but also helps to promote the improvement of digital media teaching effectiveness.

## Data Availability

The datasets used during the current study are available from the corresponding author on reasonable request.
